# Tribological Performance of Graphite Nanoplatelets Reinforced Al and Al/Al_2_O_3_ Self-Lubricating Composites

**DOI:** 10.3390/ma14051183

**Published:** 2021-03-03

**Authors:** Emad Omrani, Afsaneh Dorri Moghadam, Ashish K. Kasar, Pradeep Rohatgi, Pradeep L. Menezes

**Affiliations:** 1 Department of Materials Science and Engineering, University of Wisconsin-Milwaukee, Milwaukee, WI 53201, USA; eomrani@uwm.edu (E.O.); afdorri@gmail.com (A.D.M.); 2 Department of Mechanical Engineering, University of Nevada, Reno, NV 89557, USA; akasar@nevada.unr.edu

**Keywords:** graphite nanoplatelets, wear rate, self-lubricating composite

## Abstract

In the present work, the effect of graphite nanoplatelets (GNPs) on tribological properties of the aluminum (Al), and Al/alumina (Al_2_O_3_) composite are studied. GNPs are multilayer graphene sheets which were used as a solid lubricant material. Two sets of composites, Al/GNPs and Al/GNPs/Al_2_O_3_ with varying amounts of reinforcements, were synthesized by powder metallurgy that involves cold compaction followed by hot compaction. The hardness of the composites increased with the addition of GNPs and Al_2_O_3_. The Al/GNPs composite with 1 wt.% of GNPs (Al/1GNPs) showed a 20% increase in hardness whereas Al/GNPs/ Al_2_O_3_ composite with 1 wt.% GNPs and 2 wt.% Al_2_O_3_ (Al/1GNPs/2Al_2_O_3_) showed 27% increases in hardness compared to the pure Al. The coefficient of friction measured at 20 N was observed to be 22% and 53% lesser for Al/1GNPs and Al/1GNPs/2Al_2_O_3_, respectively, compared to corresponding alloys without graphene Al. The X-ray diffraction and scanning electron microscopy analysis revealed the presence of GNPs at the worn surface after the tribology tests. The wear rate was also reduced significantly. In comparison with pure Al, the Al/1GNPs and Al/1GNPs/2Al_2_O_3_ composites resulted in 5- and 20-times lesser wear rate, respectively. The addition of Al_2_O_3_ caused reduction in wear rate due to higher hardness and load carrying ability, whereas composites with more than 1 wt.% GNPs showed higher wear rate due to lower hardness and higher porosity. The Al/1GNPs/2Al_2_O_3_ composite exhibited the least coefficient of friction (0.2–0.25) and wear rate (1 × 10^−6^–4 × 10^−6^ mm^3^/N.m) compared to other GNPs and Al_2_O_3_ reinforced Al composites. The worn surfaces were further analyzed to understand the wear mechanism by Raman spectroscopy, transmission electron microscopy, and x-ray diffraction to detect the Al_4_C_3_ phase formation, chemical bonding, and defect formation in graphene.

## 1. Introduction

The current demand to fulfil the requirement of high-performance materials is challenging with existing metals and alloys as they have limited properties. When metals are reinforced with suitable reinforcements, the manufactured metal matrix composites (MMCs) contain improved properties that can be optimized to have a wide range of properties such as being lightweight, high specific strength, high hardness, and superior tribological properties. Generally, it has been observed that MMC’s properties improve with a decrease in the size of the reinforcement [1,2]. The current technologies allow production of particles below 100 nm that can be used as a reinforcement to improve desired properties. Accordingly, a metal matrix composites reinforced with nanosized particles are termed as a metal matrix nanocomposite (MMNCs). The addition of nanoparticles can lead to overcome of the shortcomings of composites such as poor ductility, machinability, and lower fracture toughness. Generally, the size of reinforcement influences mechanical properties, such as strength, ductility, and fracture toughness of the MMCs. Therefore, the nano sized reinforcement can be advantageous [1]. 

Carbonaceous materials, such as graphite, graphene and carbon nanotubes (CNTs), and graphene are suitable reinforcement for metal matrices [3,4]. Among these carbon materials, graphene is an excellent candidate for reinforcement due to its excellent mechanical properties, mainly high fracture strength (125 GPa), and Young’s modulus (~1100 GPa) [5], making it a singular reinforcement option for any metal matrix including aluminum, copper, and its alloys [6]. In addition to superior strength, graphene has higher thermal conductivity (~5000 Wm^−1^ K^−1^) and low density (1.06 g cm^−3^), making it an ideal reinforcement material for metal (e.g., aluminum) [7]. In fabrication of MMCs, graphene nanosheets are widely used which are stacks of graphene platelets (5–100 nm) also known as graphite nano platelets (GNPs) [8,9,10,11]. Also, single graphene layer is challenging to disperse uniformly in its free state. In addition, multi-layer graphene (3–4 layers) has shown excellent wear resistance against steel for 47,000 cycles at 0.5 GPa contact pressure compared to single-layer graphene that lasted for 6400 cycles [12]. Therefore, the wear resistance property of graphene stack or GNPs makes it a suitable reinforcement for self-lubricating MMCs.

Aluminum alloys and composites are widely used materials due to their high strength to weight ratio, superior corrosion and tribological properties [13,14]. Particularly, aluminum/graphene composites show enhanced tribological performance to due to its wear mechanism to form tribofilm. The low yield stress of aluminum alloys causes extensive deformation at the sliding interface while the graphene in aluminum/graphene composite forms a protective, stable tribofilm [15,16]. The tribofilm hinders direct metal to metal contact and hence reduces friction and wear. For example, AA6061-graphene composites with different graphene content synthesized by spark plasma sintering showed the lowest coefficient of friction (COF) for 10 and 15 wt.% graphene compared to 2 and 5 wt.% graphene content [17]. It is due to the formation of continuous graphene tribofilm at the interface observed on the worn surface. However, there was no significant improvement in the wear rate for the composite compared to unreinforced Al6061. Also, the hardness and Young’s modulus were reduced by 17% and 45%, respectively, by adding 10 vol.% of graphene, respectively. In our previous study on pure Al-GNP composite, we found a lower COF with 1 wt.% GNPs in the Al matrix compared to unreinforced Al [18]. However, the wear rate was higher, and hardness was lower for the composite. The unreinforced Al showed a hardness value of 92.48 ± 0.45 HRF while the addition of 1 wt.% graphene resulted in a lower hardness value of 86.08 ± 0.58 HRF for the composite. The higher wear rate of 1 wt.% of graphene composites was due to lower hardness. The degradation of mechanical property in Al was also observed by other studies due to porosity and agglomeration of graphene [19]. To overcome the issues of non-uniform distribution of graphene in the metal matrix, different ways are discussed in the review article [20] that includes graphene coating of metal powder by chemical vapor deposition prior to compaction and laser sintering of graphene-metal powders. These synthesis techniques have been performed on the copper-graphene composite [21] and nickel-graphene composite [22]. However, these methods are not cost-effective. 

The addition of GNPs is mainly aimed to develop self-lubrication property in the metal matrix, and as discussed, the addition of GNPs has also led to a reduction in yield strength and hardness. Specially, a higher amount of GNPs aimed to provide a continuous supply of graphene at the contact surface can cause agglomeration of graphene and reduce the strength. The alternate way to fully utilize the lubricious properties of GNPs is by the addition of second hard nanoparticles, such as oxides and carbides. In our previous studies, we have shown that the addition of hard particles, such as Al_2_O_3_, TiO_2_, and TiB_2_ in Al matrix, provided improved mechanical and tribological properties [23,24]. The mechanical properties of the Al-based composite with graphene and Al_2_O_3_ reinforcement have been studied. Kumar et al. [25] investigated the mechanical properties of the AA2024 and AA2219 alloys reinforced with 8 wt.% Al_2_O_3_ and varying amounts of graphene (0.25, 0.5, 0.75, and 1 wt.%). The composites were prepared by hot compaction. The hardness value of the composite increased with increase in graphene content, whereas the maximum compressive stress was observed for 8 wt.% Al_2_O_3_/0.75 wt.% graphene for both the Al alloy matrix. However, this work was aimed to evaluate the mechanical properties, and Al_2_O_3_ content was not varied. Using a different route, Zhang et al. [26] synthesized the graphene/Al_2_O_3_ reinforced Al composite inspired by nature where graphene/Al_2_O_3_ acts as hard bricks for load-bearing in the Al matrix. The authors used Al flakes and graphene oxide nanosheets and manufactured the composite firstly by the formation of graphene oxide-Al foam followed by compaction and sintering for densification. During sintering, Al reacted with graphene oxide to form Al_2_O_3_. The structure showed a significant increase in hardness (210%) and stiffness (78%) compared to pure Al. However, this synthesis technique does not show the amount of Al_2_O_3_ formed within the composite. Also, due to compaction of the graphene-oxide/Al foam can lead to severe defects in the graphene sheet and affect the performance. 

The addition of GNPs along with hard nanoparticles of Al_2_O_3_ in the Al matrix requires optimization of the manufacturing process to avoid the formation of undesirable phases, amount of reinforcement materials, and their role. To further develop the understanding of these nano reinforcements, a systematic study of tribofilm is required using a sophisticated instrument, such as a transmission electron microscope, that can evaluate the stability of graphene tribofilm.

Looking at the current state of knowledge and the shortcomings in the existing research streams, Al/GNP/Al_2_O_3_ composites were synthesized by a combination of cold and hot compaction of ball-milled powders and tribological tests were conducted under dry sliding conditions. The effect of reinforcement was studied by keeping the maximum amount of reinforcement to 3 wt.%. After tribological tests, the wear track and tribofilm were characterized by SEM, TEM, and Raman spectroscopy to understand the wear mechanisms, presence of graphene at the tribofilm, and detection of new phase formation. The roles of GNPs and Al_2_O_3_ are discussed on the tribological performance of the composites.

## 2. Experimental Procedure 

### 2.1. Composite Synthesis 

Aluminum composites were synthesized by varying the GNPs and alumina weight percentage. The compositions are listed in Table 1. For hybrid composites that consist of GNPs and alumina, the summation of wt.% of reinforcement is kept 3% by increasing the graphene content from 1 to 3 wt.% while adjusting the alumina wt.%.

The composites were prepared by powders of pure Al (particle size: 75 μm, Acros Organics, Waltham, MA, USA), Al_2_O_3_ (particle size: 47 nm, Nanophase, Romeoville, IL, USA), and GNPs (thickness: 10 mm, diameter: 1–2 μm, Asbury, Asbury, NJ, USA). Before the synthesis of composite, GNPs were ball milled for three hours to reduce the thickness. First, reinforcement slurry was prepared by mixing and ultrasonication of the as-received Al_2_O_3_ nano powder dispersed in 99.9% benzene and ball-milled GNPs. Then, the prepared slurry and Al powder were added to a planetary ball mill and milled at 500 rpm using steel balls with ball-to-powder weight ratio of 10:1 for 6 h. Two different diameter steel balls, 20 and 5 mm, were used with a 2:1 ratio. The milled composite powders were dried at 135 °C for 1 h to remove the benzene in the vacuum oven to prevent the oxidation of powders. After drying, the powders were first compacted at room temperature in a steel die at compact pressure of 200 MPa. After cold compaction, hot compaction was performed at 525 °C with a compact pressure of 500 MPa in air for 5 min. The resulting cylindrical samples were 25.4 mm in diameter and 10 mm thick.

### 2.2. Characterization Techniques

Comprehensive characterization tests were designed for the synthesized composites to understand the relationship between processing, microstructure, and performance using the following techniques. Prior to the synthesis of composites, a field emission scanning electron microscope (SEM) (Hitachi S-4800, Hitachi, Tokyo, Japan) was used to characterize the Al powders, GNPs, and alumina nanoparticles. The micrograph of the composites was captured using scanning transmission electron microscopy (FEI/Tecnai^TM^ F30 300 kV TEM) along with a high angle annular dark field (HAADF) detector. X-ray diffraction (XRD) data on the milled powder and bulk samples performed by a D8 Bruker diffractometer which used Cu Kα1 radiation (λ = 0.15406 nm). The XRD scans were performed from 2θ = 15° to 2θ = 85° with a step size of 0.02° and counting time of 0.3 s/step). After the tribology test, the worn surfaces of the samples were investigated to understand the wear mechanism under SEM (JEOL JSM-6460 LV, JEOL, Tokyo, Japan). The formation of tribofilm on the worn surfaces was confirmed by Raman spectra, obtained by Renishaw Inc. 1000B Raman spectroscopy using a red Helium neon laser. Tribo surfaces were analyzed by transmission electron microscope (TEM) using Phillips CM-200 operating (Philips, Amsterdam, The Netherlands) at 200 kV. The TEM thin films were prepared by A FEI^TM^ TEM200 Focused Ion Beam with Gallium ion source.

### 2.3. Hardness Test

The hardness of the composites was measured by the standard Rockwell Hardness- B scale (HRB) using a steel ball of 1.588 mm diameter. Five independent hardness measurements were carried out to calculate the average and standard deviation values. 

### 2.4. Tribology Tests

The tribological properties of the synthesized composites were evaluated using pin-on-disk tests on Rtec MFT-5000 tribometer. All the pin-on-disk tests were conducted in dry condition. In the tests, the pins were prepared out of the synthesized composites, 6 mm in diameter and 8 mm in height. The contact surface of composite pins was kept flat. The 440C stainless steel disks with a hardness of 62 HRC were prepared to act as the counter body in the wear test. The commercial 440 stainless steel (C: 0.95–1.2%, Cr: 16–18%, Mn: 1%, Si: 1%, Mo: 0.75%, *p* < 0.04%, S < 0.03%) was machined to make disks of 55 mm diameter and 10 mm thick. The experiments were conducted at different normal loads of 5, 10, 15, and 20 N with a constant sliding speed of 25 mm/s (or 120 rpm at 40 mm wear track) for a total sliding distance of 1.5 km. Before the pin-on-disk tests, the pin and disk surfaces were cleaned using acetone. During the tests, coefficient of friction (COF) and linear wear loss were recorded. Before SEM analysis of worn surfaces, the pin and disk specimens were cleaned using acetone and then hexane.

The average COF value was calculated for a complete sliding distance of 1.5 km. The linear wear-loss was recorded using a linear variable differential transducer. The recorded linear wear loss of each pin was used to calculate the volumetric wear loss (V, mm^3^) using Equation (1) as per the geometry of a cylindrical pin.

(1) $$V={\;\pi r}^{2}.h$$

where r is the pin radius, and h is the linear displacement. For each testing condition, three repetitive tests were performed, and the results of the average of three tests were reported. 

## 3. Results and Discussion

### 3.1. Microstructure

**Figure 1:**
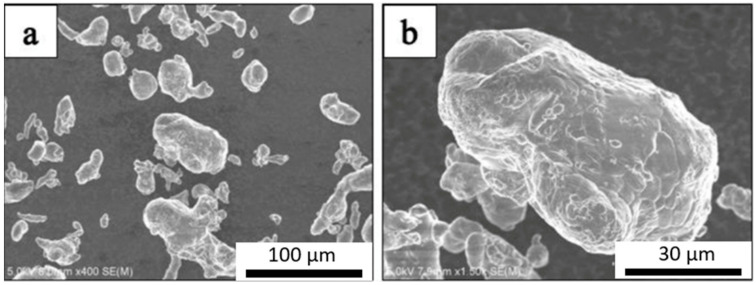
SEM micrograph of Al powders at different magnifications (**a**) 400× and (**b**) 1500×.

**Figure 2:**
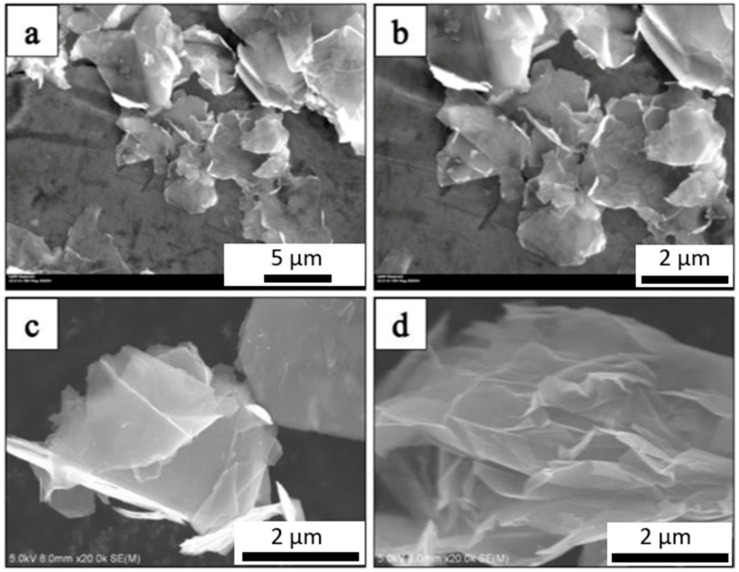
SEM micrograph of graphite nanoplatelets (GNPs) at different magnifications (**a**) 3000×, (**b**) 5000×, (**c**,**d**) at 20,000×.

**Figure 3:**
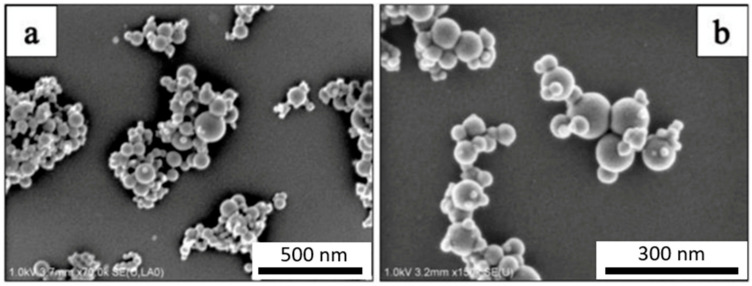
SEM micrograph of Al_2_O_3_ nanoparticles at magnification of (**a**) 70,000× and (**b**) 150,000×.

**Figure 4:**
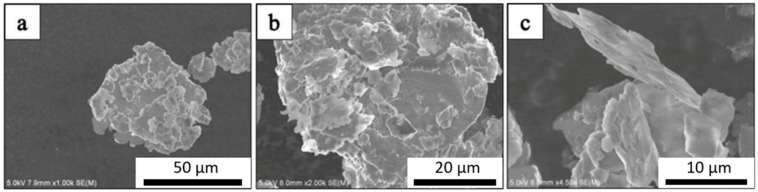
Flake shaped morphology of (**a**) pure Al, (**b**) pure Al and (**c**) Al-1%GNP after 6 h of ball milling.

The SEM micrograph of the as-received Al powder is shown in Figure 1. The micrographs confirm that the average particle size of the Al powder was approximately 75 μm. Figure 2 shows the SEM micrograph of GNPs. The micrographs show the nanoplatelet shape of GNPs. The micrographs of the alumina particles are shown in Figure 3, reflecting the average particle size of alumina particles of ~47 nm. The micrographs of the milled powders are shown in Figure 4, showing a flake shaped morphology caused by plastic deformation during ball milling.

**Figure 5:**
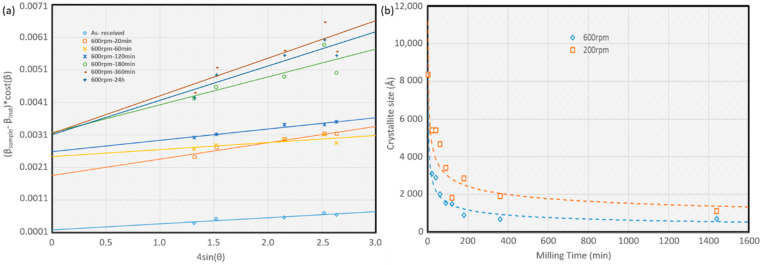
(**a**) Williamson-Hall plot of as-received Al, ball-milled Al at 200 rpm, ball-milled Al at 600 rpm, and attritor milled Al. (**b**) Variation of crystallite over the milling time at 200 and 600 rpm.

Crystallite size is an important parameter that influences the properties of the composite that can be controlled by ball milling parameters. Crystallize size after different ball milling velocity and time were calculated by Williamson-Hall plot, as shown in Figure 5a using the y-intercept of the fit. Figure 5b shows the variation of crystallite size as a function of milling time and milling rpm. It can be observed that the high energy ball mill at 600 rpm was the most effective in reducing the crystallite size in Al powder. Based on the results in Figure 5b, the selected optimum milling time was 180 min at 600 rpm to obtain the smallest crystallite size.

**Figure 6:**
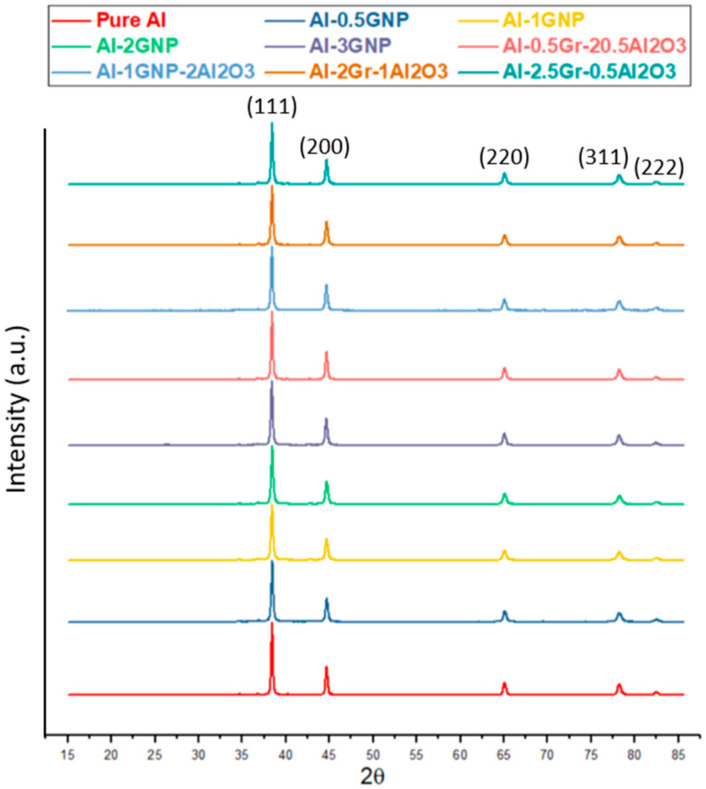
XRD spectrum of ball milled powders.

**Figure 7:**
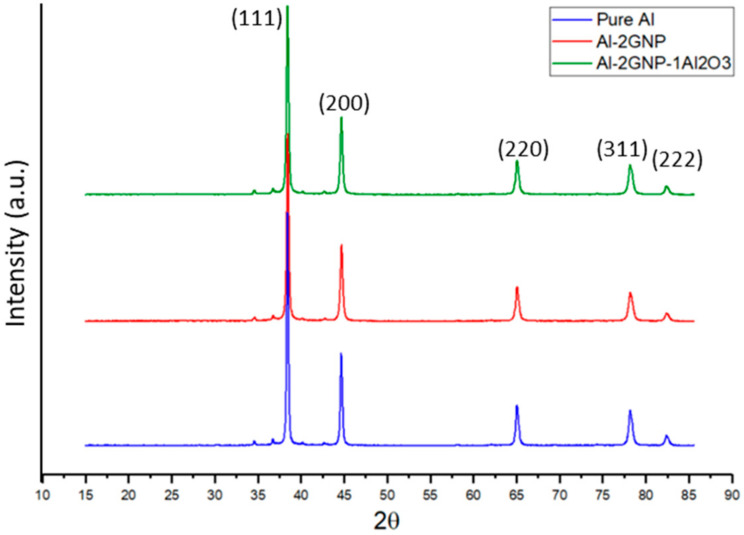
XRD spectrum of pure Al, Al-2GNP, and Al-2GNP-1Al_2_O_3_ composite.

After ball milling at 600 rpm for 180 min, XRD analysis was carried out on the milled powders to confirm the phases, and the XRD spectrums are shown in Figure 6. In all the XRD spectrums, there were five major Al peaks observed at 38.4° (111), 44.6° (200), 65.0° (220), 78.2° (311), and 82.4° (222). Figure 7 presents the XRD spectrum on selected bulk samples of pure Al, Al-2GNP, and Al-2GNP-1Al_2_O_3_ composites. The observed XRD results of the bulk samples were the same as the milled powder samples which showed five major peaks of Al. The small minor peaks appearing at 2θ equal to 34.6°, 36.76°, 40.2°, and 42.68° were that of Fe_3_O_4_. These minor peaks of Fe_3_O_4_ suggest the introduction of iron during ball milling because of the steel balls. Moreover, these XRD results confirm that during synthesis of the composites (cold and hot compaction), there is no traceable undesirable reaction or formation of new phases such as aluminum carbide. The typical aluminum carbide peaks should be observed 2θ = 31.8°, 55.0°, and 72.5° [19] which were not present here; this suggests that significant amounts of aluminum carbide does not form.

**Figure 8:**
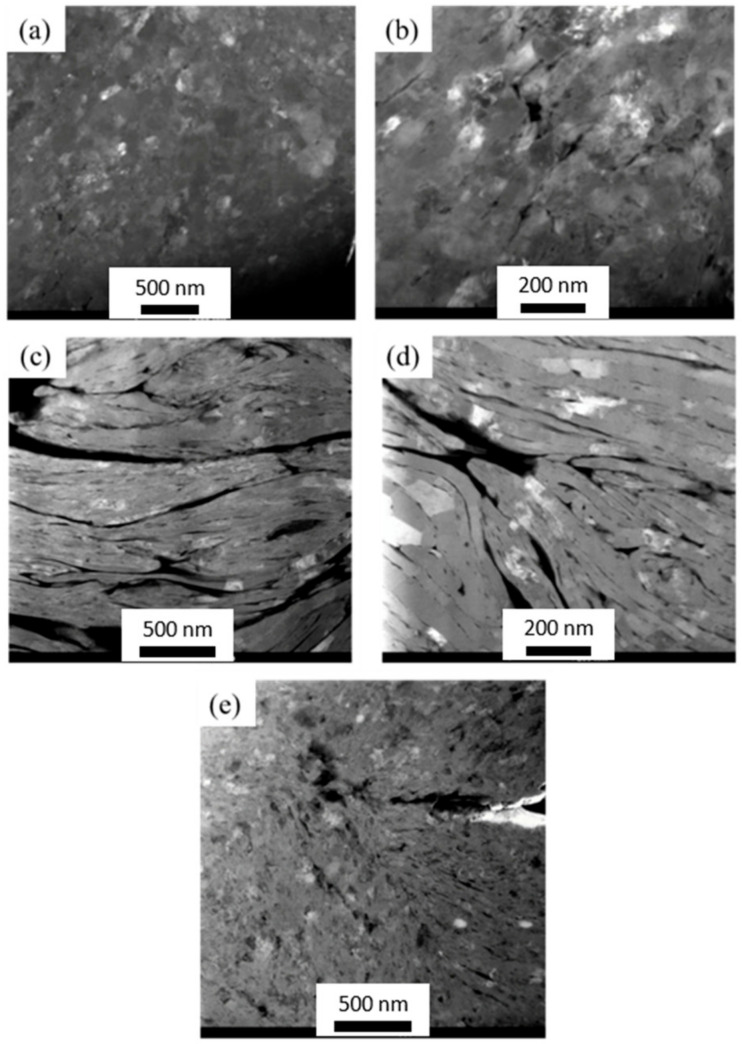
High angle annular dark field (HAADF) micrographs of (**a**,**b**) Al/1GNPs; (**c**,**d**) Al/3GNP; and (**e**) Al/2GNP/1Al_2_O_3_ composites.

Figure 8 shows the HAADF micrographs for composites. Micrographs of Al/3GNPs powder show the presence of porosity/holes, as shown in Figure 8c,d. Compared to Al/3GNPs composite, Al/1GNPs (Figure 8a,b) and Al/2GNP/1Al_2_O_3_ (Figure 8e) showed lesser porosity. The micrographs suggest higher porosity for composites with 2 and 3 wt.% GNP compared to 1 wt.% GNP. 

### 3.2. Mechanical Properties

**Figure 9:**
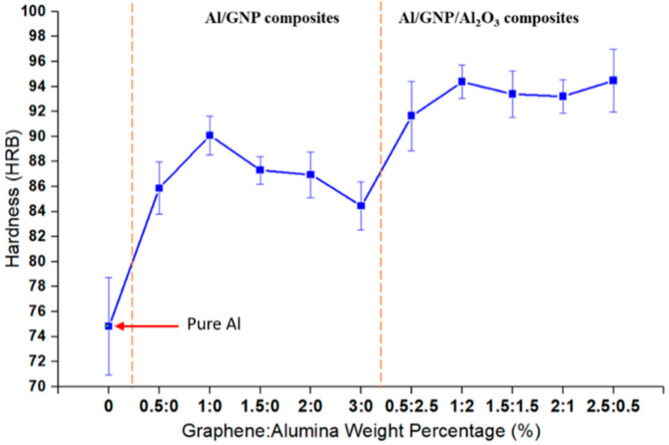
Hardness variation of Al composites with varying amount of GNPs and Al_2_O_3_.

Figure 9 shows the hardness variation for pure Al and Al composites reinforced with different amount of GNPs and alumina. Results clearly show that reinforcements increase the hardness of aluminum composites. Amongst composites, hybrid composites reinforced by GNPs and alumina have higher hardness than aluminum composites reinforced only with GNPs.

For Al/GNPs composites, the hardness value initially increases with increasing GNPs content and then decreases. Results show that with the incorporation of 0.5 wt.% GNPs in the Al matrix, the mean hardness value increased by ~15% compared with pure Al, and it further increased up to 1 wt.% GNPs. Similar results were observed by Bustamante et al. [27], who reported absence of any graphene clustering in the composite with 1.0 wt.% of graphene. Composites with more than 1 wt.% GNPs content show more porosity, which has a negative effect on the hardness of composites if one compares the microstructure of Al/1GNPs and Al/3GNPs composites in Figure 8a–d, respectively. It is evident that composite reinforced by 1 wt.% of GNPs has less porosity. Hence, the optimum weight percentage of GNP to increase the mechanical properties of Al is 1 wt.% where the hardness increased from 74.8 HRB for pure Al to 90.1 HRB for Al/1GNPs composite, which is a 20% improvement in hardness under identical experimental conditions. 

For Al hybrid composites, there is a synergetic effect of GNPs and alumina particles to enhance mechanical properties [23,24]. In a comparison between Al/GNPs and Al/GNPs/Al_2_O_3_, with the same wt.% of GNPs, the hardness of hybrid composites is higher due to hard alumina particles. The hardness value increased from 90.1 HRB for Al/1GNPs to 94.4 HRB for Al/1GNPs/Al_2_O_3_. It is also due to the grain refinement of matrix due to nanoparticles [28] and the influence of nanoparticles on strengthening of the composite as alumina nanoparticles act as obstacles to the motion of dislocation, and the Orowan mechanism is dominant [29,30].

### 3.3. Tribological Properties

**Figure 10:**
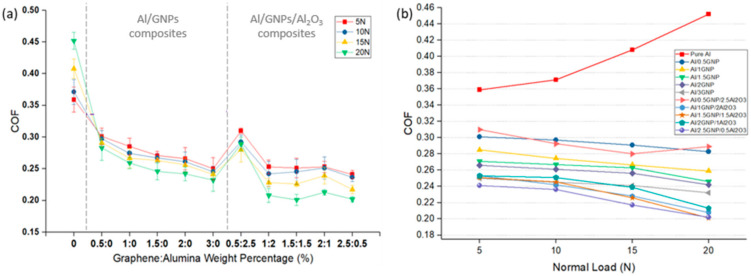
(**a**) Coefficient of friction (COF) at different loads for aluminum and its composites. (**b**) Effect of normal load on COF of various aluminum composites.

Figure 10a exhibits the COF values of Al and its composites. Results show that adding GNPs in content as low as 0.5 wt.% can decrease COF significantly. The reduction in COF of the Al/GNPs composites with increased GNPs content is generally expected. It is evident in the figure that embedding GNPs can decrease the COF of Al composite. Multilayer graphene or GNPs is a solid lubricant which promotes the formation of lubricious tribofilm at the sliding interface, therefore reducing the surface-to-surface contact. This conclusion is consistent with that of aluminum/graphite composites reported by other studies [31,32,33,34]. Although 0.5 wt.% of GNPs decreases the COF of the composite with respect to pure Al, the incorporation of 0.5 wt.% GNPs may not be enough to let the solid lubricant be continuously available at the sliding interface. The composites with higher GNPs content (2 and 3 wt.%) showed further decrease in COF. It is due to a continuous supply of graphene at the contact surface that COF is reduced through the lubricating tendency of GNPs. 

The effect of load on the COF of Al and its various composites is shown in Figure 10b. In unreinforced Al, results show that COF increases with increasing the load due to plowing action. On the contrary, in Al/GNPs and Al/GNPs/Al_2_O_3_ composites, there is a minor decrease in the COF with increasing loads. The possible explanation for this phenomenon may lay in the lubricating behavior of the reinforcement and the fact that higher wear releases more solid lubricants into the contact area. In other words, when a normal load is higher, substantial wear initially occurs. Consequently, the higher volume of worn composites releases a higher amount of alumina and lubricating graphene mixture onto the contact surface. Therefore, these released particles can form a protective tribofilm.

**Figure 11:**
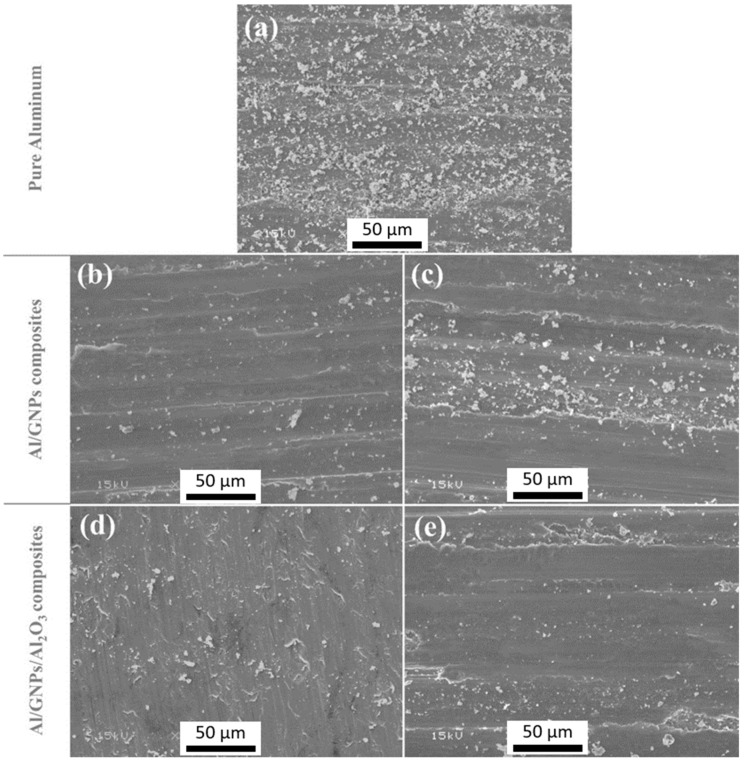
Worn pin surfaces of (**a**) pure aluminum, (**b**) Al/1GNPs, (**c**) Al/3GNPs, (**d**) Al/1GNPs/2Al_2_O_3_ and (**e**) Al/2GNPs/1Al_2_O_3_ after pin-on-disk tests at 15 N.

In comparison between Al/GNPs composites and Al hybrid composites, it is obvious that the COF of Al/GNPs/Al_2_O_3_ composites is lesser than Al/GNPs composites except for Al/0.5GNPs/2.5Al_2_O_3_. The lower COF of hybrid composites is due the hard alumina particles. This was confirmed by the SEM of the wear track generated at 15 N, as shown in Figure 11. The grooves can be clearly seen in pure aluminum and Al/GNP composites in Figure 11, which occurs due to the plowing of the surfaces. The composites with hard alumina particles show lesser grooves (Figure 11d,e) that suggest a lesser plowing component of friction for hybrid composites. Thus, overall COF is reduced.

**Figure 12:**
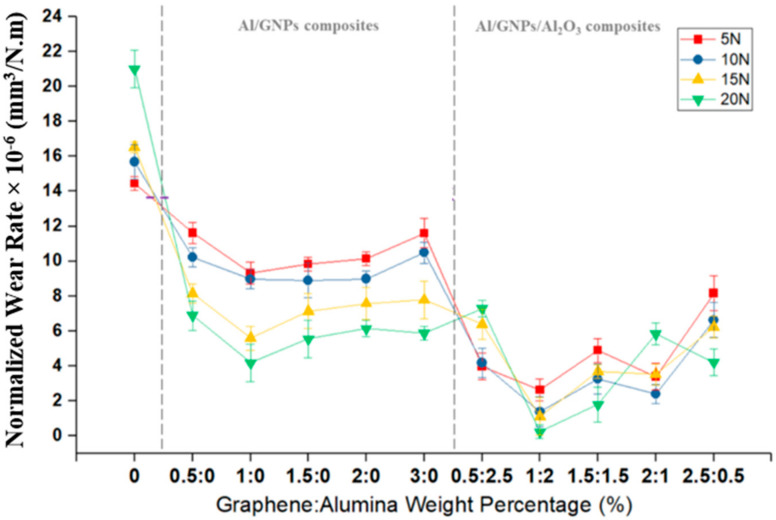
The variation of wear rate for Al and its composites at different loads.

Wear resistance of Al and its composites are plotted in terms of normalized wear rate. The normalized wear rate is equal to wear volume divided by the total sliding distance and normal load. Figure 12 shows the effect of reinforcement types and the wt.% of reinforcement on the wear rate. The pure Al showed the highest wear rate compared to all the composites due to the presence of GNPs and alumina embedded in the Al. Consequently, the lifetime of the Al is significantly prolonged as the sliding surface is continuously supplied with lubricating particles. The hardness of the material is a deciding factor in explaining the wear behavior of materials; as per the Archard equation [35], the wear rate is increase as hardness of the material decreases. For this reason, Al/1GNPs and Al/1GNPs/2Al_2_O_3_ show the lowest wear rate among Al/GNPs and Al/GNPs/Al_2_O_3_, respectively, compared to pure Al and other composites. Therefore, as expected from the Archard equation, the wear rate decreases by increasing GNPs up to 1 wt.% due to an increasing the hardness. Besides, the GNPs form a lubricant protective tribofilm on the worn surfaces, which consequently reduces the wear rate by minimizing the direct contact between two mating surfaces. As seen earlier, adding more GNPs, above 1 wt.%, has a negative effect on hardness, possibly due to agglomeration, which in turn lowers the wear resistance. In conclusion, under GNPs contents up to 1 wt.%, the composites outperformed the pure Al, while the wear performance of the composite deteriorated at high GNPs contents of 3 wt.%. In addition, there is another effect of GNPs which improves the wear properties, and that is the ability of GNPs to protect the surface against oxygen and enhancing oxidation resistance. In the presence of oxygen, Al will form a thin oxide layer due to oxidation that can act as a third body abrasive during sliding.

By comparing the wear rate of GNPs composite with hybrid composites, it is obvious that the wear rate of self-lubricating Al/GNPs/Al_2_O_3_ composites is lesser than Al/GNPs composites. It is due to the higher hardness of hybrid composites compared to Al/GNPs that the ability of materials for lower surface damage is enhanced, as shown by wear track profile in Figure 11. For Al/GNPs and Al/GNPs/Al_2_O_3_ composites with the same weight percentage of GNPs, hybrid Al composites have a lower wear rate due to the synergetic effect of GNPs and alumina. Alumina particles provide hardness and GNPs form lubricious tribofilm. The incorporation of hard alumina nanoparticles in Al matrix resists the plowing action of the counter steel and reduces the wear. However, the alumina particles above a critical amount in the Al matrix can act as a third body abrasive and cause severe wear. Thus, the optimized amount of alumina is required. On the other side, less than the optimum amount of alumina cannot provide enough resistance or hardness to avoid groove formation from the counter body. In the present study, the optimum amount of alumina is 2 wt.%, and Al composites reinforced with 1 wt.% GNPs and 2 wt.% alumina exhibit the lowest value of wear rate. The wear rates of Al/1GNPs composite are 9.3, 9.0, 5.6, and 4.2 mm^3^/N.m × 10^−5^ at 5, 10, 15, and 20 N, respectively which are 72%, 85%, 80%, and 94% higher than the wear rate of Al/1GNPs/2Al_2_O_3_ composites (2.6, 1.4, 1.1, and 0.2 mm^3^/N.m × 10^−5^ at 5, 10, 15, and 20 N, respectively). Moreover, the wear rate of Al/1GNPs/2Al_2_O_3_ composites is 80–95% lesser than the wear rate of pure Al at the applied normal load range. 

**Figure 13:**
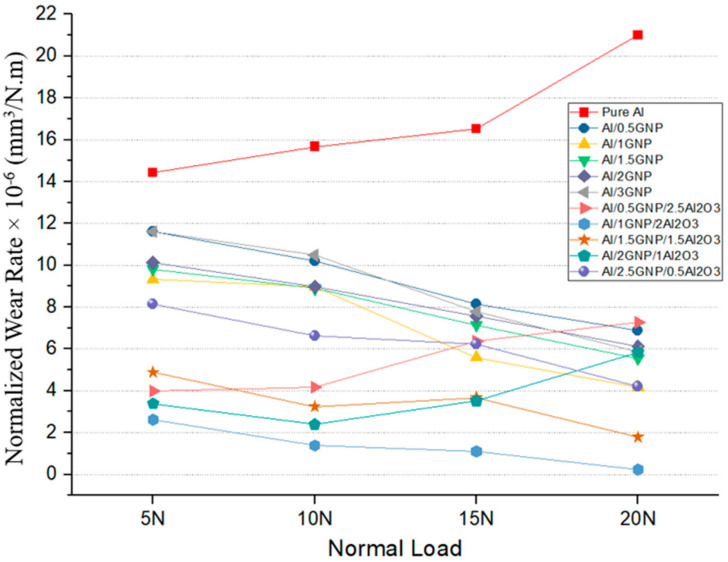
Effect of normal load on the wear rate of aluminum composites.

The effect of normal load on the wear rate of pure Al and composites is shown in Figure 13. The same trend of COF can be observed for wear rate where the wear rate of pure Al was increased by increasing the load while, on the contrary, the wear of composites, including the Al/GNPs and Al/GNPs/Al_2_O_3_, decreases with increase in the normal load. The reason for lower wear at higher load is that the formation of tribofilm due to initial wear protects the surface and stops continuous wear during sliding. Thus, the total wear at the end of the test comes out to be lower than pure Al.

### 3.4. Chemical Characterization of Tribofilm

**Figure 14:**
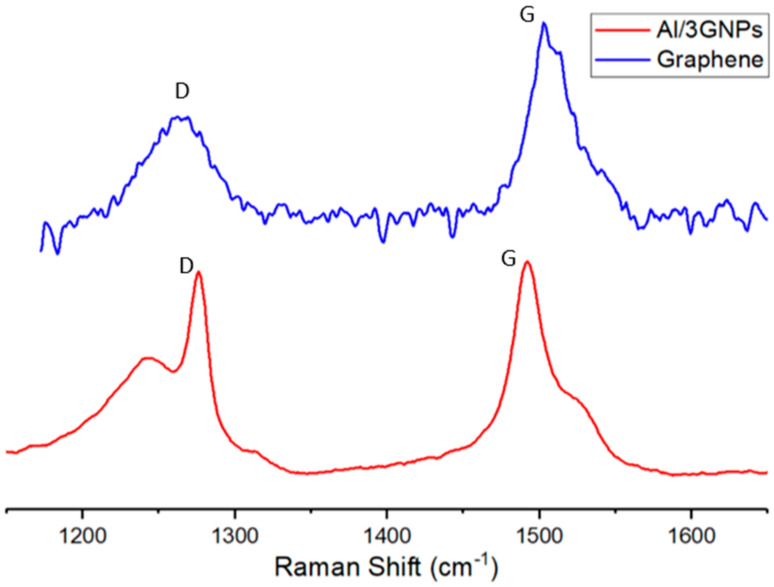
D- and G-band Raman spectrum of initial GNPs powder and Al/3GNPs composites.

**Figure 15:**
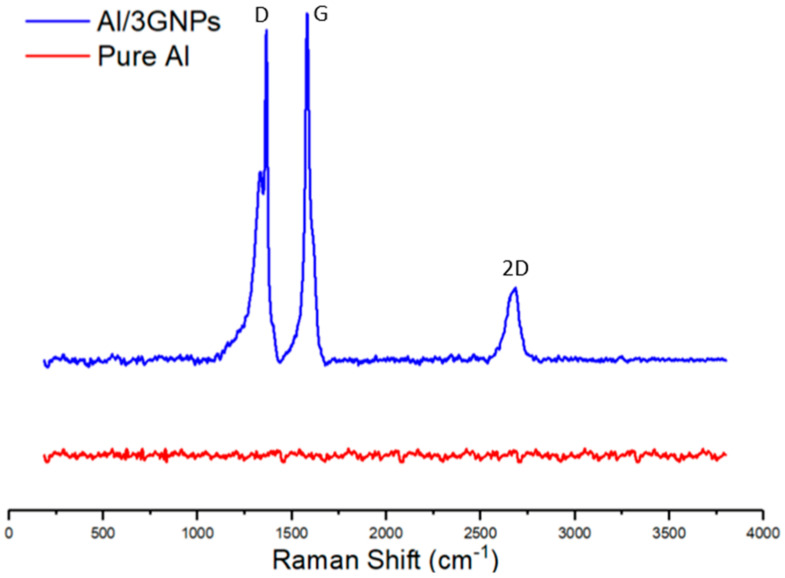
The Raman spectrum of worn surface of Al/3GNPs composite.

**Figure 16:**
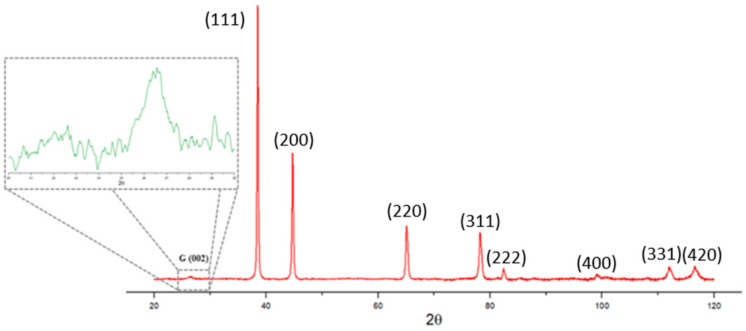
XRD of worn surface of Al/3GNPs composites at 20 N.

After the pin-on-disk experiment, the tribofilm formed at contacting surfaces were analyzed by Raman spectroscopy and TEM. Figure 14 compares the D- and G-band Raman spectrum of initial GNPs powder and Al/3GNPs composites. G band is a characteristic of graphene, whereas D band occurs due to distortion or defects in the graphene [36]. The ID/IG ratio in the Raman spectrum increased significantly after milling, as shown in Figure 14. The full Raman Spectra of worn surface of Al/3GNPs composite is shown in Figure 15. The D band and the secondary peaks appear at the edge of the D band are defect-induced Raman features. Therefore, milling and wear induced more defects in GNPs because of the physical force applied during the processes. The ratio of ID/IG is inversely proportional to the crystallite size of graphene [37]. The increase in ID/IG after milling and wear tests also implies that the crystallite size of graphene is decreased. On tribofilm of Al/1GNP/2Al_2_O_3_ composite, GNPs spectrum could not be detected due to lesser GNP. However, a similar behavior of defects generation is expected. In addition, the XRD results of worn surface of Al/3GNPs, shown in Figure 16, reveal the presence of GNPs on the worn surface by the appearance of (002) peak at 26.6°. As mentioned for Raman spectrum, XRD also could not capture GNP peak on Al/1GNP/2Al_2_O_3_ composite due to limited GNPs content.

**Figure 17:**
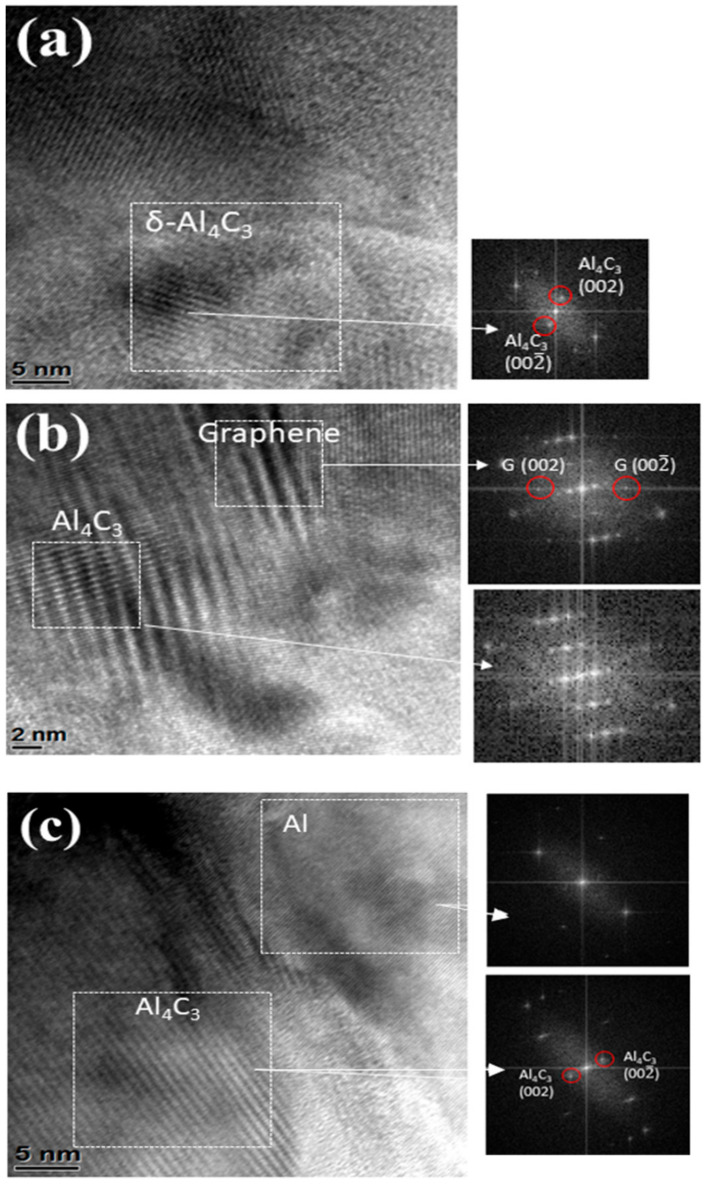
TEM image of tribo surface of (**a**) Al/1GNPs, (**b**) Al/3GNPs, and (**c**) Al/2GNPs/1Al_2_O_3_ composites.

Prior to wear test, the observed Raman spectrum (Figure 14) does not show Al_4_C_3_ peaks that indicate that Al is not reactive to GNPs during fabrication of the composites. However, the tribofilm seems to contain some Al_4_C_3_ (traceable by Raman at the interface and untraceable by the X-ray diffraction) after the wear test. This suggests that Al reacts with GNPs during wear tests and forms Al_4_C_3_. This can be attributed to the raised local temperature during the wear test. Additionally, the wear test process may introduce structural defects to GNPs and presence of these structural defect cause reaction with the Al matrix due to a low activation energy. The TEM image of tribo surface of composites proves the formation of Al_4_C_3_ compound at the interface of GNPs and Al, which form a tribofilm with chemical bonding. Figure 17 exhibits the TEM images of tribofilm for Al/1GNPs, Al/3GNPs, and Al/2GNPs/1Al_2_O_3_ composites.

## 4. Conclusions

Self-lubricating Al nanocomposites reinforced by GNPs and alumina were synthesized by the powder metallurgy technique and using cold and hot compaction. The effect of reinforcement was investigated in terms of observed microstructure, mechanical, and tribological properties. The microstructural studies using TEM shown the presence of GNPs in the Al matrix without any reaction products. The XRD results also indicated no undesirable reaction or formation of new phases during fabrication of self-lubricating Al-Graphene-Alumina nanocomposites. Thus, the TEM and XRD results confirm the feasibility of Powder Metallurgy and Hot-Pressing techniques and parameters in synthesizing Al composites reinforced with graphene and alumina. 

In case of Al/GNPs composites, the hardness increased initially up to 1 wt.% GNPs and then decreased. The hardness increased from 74.8 HRB for pure Al to 90.1 HRB for Al/1GNPs composite. The hardness of the composite with higher GNPs contents above 1 wt.% reduced due to pores. In the case of hybrid composites, the hardness value further increased with the addition of alumina, and the maximum hardness value of 94.4 HRB was observed for Al/1GNPs/2Al_2_O_3_ composite. 

With the addition of GNPs and alumina, friction coefficient and wear rate for all nanocomposites were studied in this paper, for all the normal loads in comparison with pure Al. For example, at 15 N of normal load, the addition of 1 wt.% GNPs in the self-lubricating composites caused a remarkable reduction in wear rate and COF value. The wear rate and COF decreased by 66% and 35% in comparison with the unreinforced Al. Similarly, in Al/1GNPs/2Al_2_O_3_, a 93% reduction in the wear rates and a 44% reduction in COF value was recorded in comparison with the pure Al. Due to the higher hardness of the composites, lower wear and friction values were observed. However, there is an optimum wt.% of reinforcement where the wear rate can be minimized; likewise, the hardness achieved can be maximized. This optimum value was obtained in 1 wt.% GNPs for Al/GNPs composites, and 1 wt.% GNPs/2 wt.% Al_2_O_3_ for hybrid composites. It is worth noting, unlike unreinforced Al, the self-lubricating composites have lower wear rate and COF at higher loads due to lower plowing action by the counterpart. 

The tribofilm on the wear track was analyzed by Raman Spectroscopy and TEM. Raman spectrum shows D- and G-band that confirms the worn surface is covered with GNPs tribofilm. In addition, in some nanocomposites containing graphene, an Al_4_C_3_ peak also exists on the worn surface, which was not present before tribological tests. TEM image of the worn surface of composites also confirms the formation of Al_4_C_3_ compound at the interface of GNPs and Al; this suggests that some aluminum carbide can form during the tribological testing of graphene containing composites.

The dominant wear mechanism to explain the improved wear characteristics in the composite is the formation of protective GNPs tribofilm that reduces subsequent wear. In addition, the GNPs structure as a stacked graphene layer provides continuous lubrication at the sliding interface. In hybrid composites, in addition to these mechanisms, alumina particles provide resistance to plowing that causes no or lesser groove formation on the surface. This will minimize the plastic deformation and, therefore, help the formation of tribofilm at the interface, successfully enhancing the tribological properties.

## Figures and Tables

**Table 1:** Compositions of Al-GNP composites.

Composite	GNPs (wt.%)	Alumina (wt.%)
Al/0.5GNPs	0.5	0
Al/1GNPs	1	0
Al/1.5GNPs	1.5	0
Al/2GNPs	2	0
Al/3GNPs	3	0
Al/0.5GNPs/2.5Al_2_O_3_	0.5	2.5
Al/1GNPs/2Al_2_O_3_	1	2
Al/1.5GNPs/1.5Al_2_O_3_	1.5	1.5
Al/2GNPs/1Al_2_O_3_	2	1
Al/2.5GNPs/0.5Al_2_O_3_	2.5	0.5

## Data Availability

The data presented in this study are available on request from the corresponding author.
